# National and subnational burden of mental disorders in Iran (1990–2019): findings of the Global Burden of Disease 2019 study

**DOI:** 10.1016/S2214-109X(24)00342-5

**Published:** 2024-11-20

**Authors:** Iran Subnational Mental Health GBD Collaborators, Leila Ghalichi, Seyed Vahid Shariat, Morteza Naserbakht, Mozhgan Taban, Mohsen Abbasi-Kangevari, Fatemeh Afrashteh, Marjan Ajami, Hossein Akbarialiabad, Sohrab Amiri, Jalal Arabloo, Hosein Azizi, Nayereh Baghcheghi, Sara Bagherieh, Saeid Bitaraf, Sharareh Eskandarieh, Fataneh Ghadirian, Ali Gholami, Pouya Goleij, Mojtaba Habibi Asgarabad, Aram Halimi, Mohammad Heidari, Farideh Iravanpour, Roxana Jabbarinejad, Morteza Jafarinia, Hamid Reza Khayat Kashani, Hamid Reza Koohestani, Mohammad-Reza Malekpour, Mahsa Mayeli, Reza Mirfakhraie, Mojgan Mirghafourvand, Soheil Mohammadi, Esmaeil Mohammadi, Abdollah Mohammadian-Hafshejani, Fateme Montazeri, Majid Motaghinejad, Shumaila Nargus, Hassan Okati-Aliabad, Mohammad Taha Pahlevan Fallahy, Shayan Rahmani, Ali Rajabpour Sanati, Vahid Rashedi, Nazila Rezaei, Mohsen Rezaeian, Reihaneh Sadeghian, Saeid Sadeghian, Amirhossein Sahebkar, Saman Sargazi, Yaser Sarikhani, Mahan Shafie, Seyed-Amir Tabatabaeizadeh, Amir Tiyuri, Seyed Mohammad Vahabi, Rohollah Valizadeh, Leila Zaki, Iman Zare, Mohammad Zoladl, Maziar Moradi-Lakeh, Afarin Rahimi-Movaghar, Ali H Mokdad, Mohsen Naghavi

## Abstract

**Background:**

Mental and behavioural disorders account for a large proportion of the burden of diseases in Iran. Identifying the pattern of change can help in policy making and provision of mental health services. We aimed to analyse the burden of mental disorders (excluding substance use disorders) in Iran at national and subnational levels with data from the Global Burden of Diseases, Injuries, and Risk Factors Study 2019.

**Methods:**

We used data from 1990 to 2019 on anxiety disorders, attention-deficit hyperactivity disorder, autism spectrum disorders, bipolar disorder, conduct disorder, depressive disorders, eating disorders, idiopathic developmental intellectual disability, schizophrenia, and other mental disorders in Iran and its 31 provinces. We calculated total disability-adjusted life-years (DALYs), age-standardised DALYs, and prevalence rates in 1990 and 2019, as well as the percentage change between these time periods.

**Findings:**

Mental disorders accounted for 1 159 410 (4·6%) of 25 007 732 all-cause DALYs in Iran in 1990 and 2 053 871 (10·3%) of 19 828 721 in 2019. Although total DALYs for mental disorders increased by 77·1% (95% uncertainty interval 76·7 to 77·6%) during this period, age-standardised DALY rate increased by 1·8% (–4·1 to 7·7%). The overall patterns of change were similar at the subnational level as the national level, although the rates differed between provinces with a highest-to-lowest ratio of 1·22 for age-standardised DALY rates in 2019.

**Interpretation:**

The increase in the burden of mental disorders in Iran is higher than the general trend in the world. The slight change in age-standardised DALYs suggests that the increase is mainly attributable to changes in the size and structure of the population. Considering the absolute and relative increase in the burden of mental disorders during the past 30 years at national and provincial levels, there is an urgent need to address the determinants of mental health and upgrade mental health services across all levels of care in Iran.

**Funding:**

Bill & Melinda Gates Foundation.

## Introduction

Mental and behavioural disorders are one of the leading causes of disability and years of life lost.^1^ In the past 30 years, a notable shift in the pattern and causes of burden of diseases has occurred in many countries and territories, moving from communicable to non-communicable diseases.^2^ Iran has also been among the countries with such a pattern of change.^3^, ^4^ Between 1990 and 2019, the global disability-adjusted life-years (DALYs) due to mental disorders increased from 80·8 million (95% uncertainty interval [UI] 59·5–105·9) to 125·3 million (93·0–163·2), and the proportion of global DALYs attributed to mental disorders increased from 3·1% (2·4–3·9%) to 4·9% (3·9–6·1%).^1^

The proportion of the disease burden due to mental and behavioural disorder could be especially large in societies with younger populations.^5^ Since the start of the 21st century, the proportion of the young population in Iran has had a rapid increase, with people aged 25–35 years having the highest share in the population pyramid,^6^ which reflects the high fertility rates observed after the Iranian revolution (in 1979) and before effective implementation of family planning programmes. In line with this shift in age demographics, there is existing evidence on an increasing trend in the prevalence of mental disorders in Iran during the past three decades. Screening surveys have estimated that the prevalence of mental disorders has increased from 21·0% in 1999 to 31·7% in 2015.^7^, ^8^ Apart from the changes in population composition, this timespan was accompanied by numerous stressors and changes in social, economic, and cultural determinants of mental health, which might have played a role in this increasing trend in the diagnosis of mental disorders.^9^ Economic deterioration, as a joint effect of poor policy making and administrative performance and devaluation of national currency, along with international unilateral sanctions by the USA or multilateral sanctions (enforced by various parties), has been a continuous stressor;^10^ it has been accompanied by an increasing gap between different socioeconomic strata. As a result, Iran has a high Gini coefficient and wealth inequality, which exists not only between different provinces of the country, but also within each province and each city.^11^

**Research in context**

**Evidence before this study**

From Feb 25, 2022, to April 10, 2023, we searched PubMed, PsycINFO, and Embase for papers on the burden of mental disorders in Iran published after 2019 to find any study published from the Global Burden of Diseases, Injuries, and Risk Factors Study (GBD) 2019 data. We used the search terms (((“Mental disorders”[Title/Abstract]) AND (Iran[Title/Abstract],)) AND (2019[Title/Abstract])) AND ((((“GBD 2019”[Title/Abstract]) OR (Disability[Title/Abstract])) OR (Prevalence[Title/Abstract])) OR (Burden[Title/Abstract])). We found no publications specifically dedicated to findings of the GBD 2019 data on mental disorders in provinces of Iran. The most recent comprehensive assessment of disease burden in Iranian provinces was published in *The Lancet* on April 23, 2022, using GBD 2019 data. Although the paper offers insights into mental disorders as a major category among other diseases, it does not have specific focus on mental disorders and their various subtypes. Another relevant study from the GBD 2019 Mental Disorders Collaborators was published in *The Lancet Psychiatry* on Jan 10, 2022, providing high-level findings and national-level estimates of mental disorders. Additionally, Effatpanah and colleagues published a study in the *Balkan Medical Journal* on March 1, 2024, focusing on national estimates of mental disorders in north Africa and the Middle East.

**Added value of this study**

Our study reveals that the proportion of mental disorders as part of the overall disease burden in Iran rose from 1 159 410 (4·6%) of 25 007 732 all-cause disability-adjusted life-years (DALYs) in 1990 to 2 053 871 (10·3%) 19 828 721 in 2019. We observed variations among provinces, with the largest increase estimated in the Zanjan and Gilan provinces. Despite a 77% increase in the total DALYs attributed to mental disorders, the age-standardised DALY rates for mental disorders and their most prevalent subtypes did not show considerable changes. In 2019, there was a highest-to-lowest ratio of 1·22 for age-standardised DALY rates of mental disorders among the provinces of Iran.

**Implications of all the available evidence**

Considering the absolute and relative increase in the burden of mental disorders in Iran and its provinces during the past 30 years, there is an urgent need to address the determinants of mental health and upgrade mental health services to improve accessibility and use of all levels of care in Iran.

Iran has 31 provinces with very different socioeconomic backgrounds, such as unemployment rates ranging from 6% to 16%, illiteracy rates from 8% to 24%, life expectancies of 72·9 years to 80·1 years,^12^ and mean annual incomes per capita from 250 million to more than 400 million Iranian rials.^13^ This evident inequality in wealth, socioeconomic factors, and health outcomes, as well as different cultures, natural resources, and infrastructures, affects the mental health status of the inhabitants. The provincial-level subnational data on the burden of mental disorders can assist planning to decrease inequality and improve resource allocation. Many countries share concerns regarding subnational disparities in disease burden that cannot be adequately addressed solely from a unified national perspective. Iran was one of the 21 countries in the world and the only country in the north Africa and the Middle East region that had subnational-level (ie, province-level) data studied in the Global Burden of Diseases, Injuries, and Risk Factors Study (GBD) 2019. In this subanalysis, our aim was to analyse the burden of mental disorders in Iran at national and subnational levels with data from GBD 2019.^3^ This manuscript was produced as part of the GBD Collaborator Network and in accordance with the GBD Protocol.

## Methods

### Overview

We present the GBD 2019 results for mental disorders (excluding substance use disorders which deserve a separate report because of their importance in the region) in Iran and its provinces for 1990–2019. GBD 2019 included subnational analyses for 21 countries, and Iran was one of them. In 2019, Iran had 31 provinces with a wide range of population sizes, from 0·6 million in Ilam to 14 million in Tehran. The number and boundaries of the provinces of Iran have changed since 1990; estimates of this report are based on 2019 classifications reconstructed retrospectively to 1990. A detailed description of the GBD 2019 methodology is available in a previous publication.^14^

### Case definitions

In GBD 2019, mental disorders—classified as a subgroup of the non-communicable diseases—included ten level 3 causes. Depressive disorders and eating disorders were each divided into two level 4 causes (appendix p 2). More details on definitions of each cause are available elsewhere.^1^, ^14^

### Epidemiological data

The estimation process was based on identifying multiple relevant data sources for each disorder. Similar to previous GBD iterations, systematic reviews were updated to capture all available epidemiological data for each of the causes. For mental disorders in Iran, all Persian and English literature was also reviewed by a group of Iranian GBD collaborators to augment the general GBD systematic review process. The Global Health Data Exchange source tool provides bibliographical information on the sources of data used for estimating each disease in any given location. To correct non-reference case definitions or measurement errors for non-fatal sequalae, the GBD team used network meta-regression on data in which two methods were assessed in the same location–time period or in the exact same population. This correction method was different from the use of reference definitions or measurement methods as part of the Bayesian meta-regression models in previous GBD iterations.^14^, ^15^

We used general methodologies for age-sex splitting of epidemiological indicators related to mental disorders. Whenever possible, estimates were further divided by sex and age based on available data. For example, if original studies provided prevalence data for a broad age group by sex and also for specific narrower age groups but combined for both sexes, we split age-specific estimates by sex through the reported sex ratio and its associated UI. To split the other combined sex estimates of indicators that had not reported narrower age-specific estimates, we used a meta-regression with Bayesian priors, regularisation, and trimming (MR-BRT) analysis. This analysis was used to estimate pooled sex ratios by age, and their associated UIs, subsequently used to disaggregate the combined sex estimates in the dataset. Studies reporting prevalence across age groups spanning 25 years or more (eg, groups aged 15–39 years and 40–69 years) were split into 5-year age groups through the prevalence age pattern estimated by DisMod-MR 2.1; this is an app specifically developed for the GBD that uses meta-regression and a Bayesian approach for disease modelling.

### Modelling strategies for specific mental disorders

Strategies used for modelling each of the mental health disorders, including the assumptions of each model, are available in the appendix of a previous publication,^14^ as well as being summarised in our appendix (pp 3–7). In general, systematically collected data for each disorder were used in combination with prior distribution, study-level, and location-level covariates to estimate epidemiological indicators for each age, sex, location, and year.

There were different types of priors for each of the disease models. As part of the general approach, data at each higher level were used as a prior for lower levels in a cascade process of estimation. This approach means that age-sex distribution of disease prevalence (and other epidemiological indicators) at global, super-regional, regional, and national levels has been used as prior distribution for the lower levels—ie, the subnational level. In addition, there were specific priors for each of the mental health disorders (appendix pp 3–5). These priors and assumptions were shaped by the following factors: definitions and diagnostic criteria of mental disorders, age constraints seen in the raw data for each parameter, and the extent to which the prior was able to guide the modelled outputs towards the raw data. Each prior underwent a thorough review in collaboration with experts in the relevant disorders.^1^

Estimates with known biases were adjusted or crosswalked accordingly before they were added to DisMod-MR 2.1 with data. For each crosswalk of interest, pairs of the reference and the alternative estimates were matched by age, sex, location, and year. This matching was done for both within (when possible) and between study pairs. These pairs were then used as inputs for the MR-BRT analysis. The MR-BRT analysis produced a pooled ratio between the reference estimates and alternative estimates, which was used to adjust all alternative estimates in the dataset. The appendix (pp 5–7) provides the list of study-level and location-level covariates used for each of the mental disorders alongside the coefficients used for adjustment of the data.

Each of the models provided a consistent set of epidemiological indices (including prevalence, incidence, cause-specific mortality, remission, and duration), and measures of these indicators were used for calculating burden after some adjustment for comorbidity.^14^

### Comorbidity adjustment

We used a simulation method to adjust for comorbidity. The co-occurrence of different causes was estimated based on independent probabilities of having each sequela in populations of 40 000 individuals for each location, age, sex, and year.

Based on the analysis of individual-level data (from the US Medical Expenditure Panel Survey, the National Epidemiologic Survey on Alcohol and Related Conditions, and the Australian National Survey of Mental Health and Wellbeing of Adults), age was by far the most important predictor of comorbidity. The analysis revealed the results adjusted for independent comorbidities cover most of the variabilities incurred by comorbidities. A correction based on a multiplicative function of disability weights based on independence gave a correction of years of life lived with disability (YLDs) in the order of two-thirds of the correction when both independent and dependent comorbidities were included; although there are well known examples of dependent comorbidity (eg, clustering of anxiety and alcohol use disorders), we did not include dependent comorbidity correction in the analysis. This decision was because of the complexity of the analysis if we had included dependent comorbidities. Based on this approach, simulants end up having none to multiple disease sequelae. For each comorbid condition, a multiplicative disability weight was calculated based on the disability weights of sequelae that they have acquired.

### Calculation of burden

In the GBD study, estimates of prevalence of diseases are used to calculate YLDs. YLDs were estimated by multiplying prevalence of each sequela by an appropriate disability weight for the same health condition. Disability weights quantified the amount of health loss associated with each sequela (or consequence of a disease or injury) in a range between 0 (perfect health) and 1 (death). These weights were derived from community-based surveys in Bangladesh, Indonesia, Peru, Tanzania, the USA, Hungary, Italy, Sweden, and the Netherlands, and an open web-based survey available in English, Spanish, and Mandarin.^14^

The years of life lost were calculated based on the estimates for disease-specific mortality rates and the standard life expectancy at the exact age of death. DALYs were calculated through the addition of YLDs and years of life lost. For the mental disorders not recognised as causes of death, YLDs approximated DALYs. The GBD standard population was used for age standardisation of the estimates; we used the non-weighted mean of proportions of different age groups from the population estimates for all national locations with a total population of at least 5 million people in 2019 to generate an updated standard population age structure.^16^ For each of the estimates, a 95% UI was derived from the 25th and 975th ordinals of the 1000 draw of the posterior distribution.

### Role of the funding source

The funder of the study had no role in study design, data collection, data analysis, data interpretation, or the writing of the report.

## Results

In 1990, mental disorders caused an estimated 1 159 410 DALYs (95% UI 851 688–1 530 200) in Iran, accounting for 4·6% (n=1 159 410/25 007 732) of all-cause DALYs. Depressive disorders were the leading cause of DALYs among all mental disorders in 1990, followed by anxiety disorders, bipolar disorder, and schizophrenia (table 1).

**Table 1:** Total DALYs and percentage change for mental disorders in Iran

	**1990 DALYs**	**2019 DALYs**	**Percentage change, 1990–2019**
**Mental disorders**	**1 159 410 (851 688 to 1 530 200)**	**2 053 871 (151 6179 to 2 714 847)**	**77·1% (76·7 to 77·6%)**
Anxiety disorders	363 830 (253 144 to 501 829)	608 056 (422 918 to 835 643)	67·1% (66·4 to 67·8%)
Attention-deficit hyperactivity disorder	22 242 (12 585 to 38 641)	26 330 (14 932 to 44 602)	18·4% (16·3 to 20·5%)
Autism spectrum disorders	34 582 (22 489 to 50 672)	47 262 (31 078 to 68 539)	36·7% (34·8 to 38·6%)
Bipolar disorder	83 936 (51 139 to 130 234)	160 226 (98 666 to 248 098)	90·9% (89·3 to 92·5%)
Conduct disorder	62 327 (35 600 to 98 602)	50 474 (28 501 to 80 429)	−19·0% (−20·0 to −18·1%)
Depressive disorders	398 046 (265 358 to 558 440)	813 441 (553 620 to 1 143 612)	104·4% (103·6 to 105·1%)
Eating disorders	25 251 (15 749 to 37 479)	50 000 (31 280 to 74 071)	98·0% (95·0 to 101·0%)
Idiopathic developmental intellectual disability	60 883 (33 590 to 97 087)	46 922 (23 606 to 79 094)	−22·9% (−23·9 to −22·0%)
Other mental disorders	39 261 (25 100 to 60 029)	93 040 (59 192 to 142 670)	137·0% (134·2 to 139·8%)
Schizophrenia	69 045 (50 674 to 88 189)	158 115 (114 789 to 202 150)	129·0% (127·0 to 131·1%)

Data in parentheses are 95% uncertainty intervals. DALYs=disability-adjusted life-years.

In 2019, the number of DALYs caused by mental disorders rose to 2 053 871 (95% UI 1 516 179–2 714 847) in Iran, accounting for 10·3% (n=2 053 871/19 828 721) of all-cause DALYs. Depressive disorders, anxiety disorders, bipolar disorders, and schizophrenia were still the leading cause of DALYs among all mental disorders (table 1). Overall, an increase was observed in the burden of disease between 1990 and 2019, except for conduct disorders and idiopathic developmental intellectual disabilities. In 2019, age-standardised DALY rates of mental disorders in Iran were higher than the global mean both in females (2624 *vs* 1703 per 100 000) and males (1976 *vs* 1427 per 100 000); the female-to-male ratio of burden was also higher in Iran than the global mean (1·34 in Iran compared with 1·19 globally).

**Figure 1:**
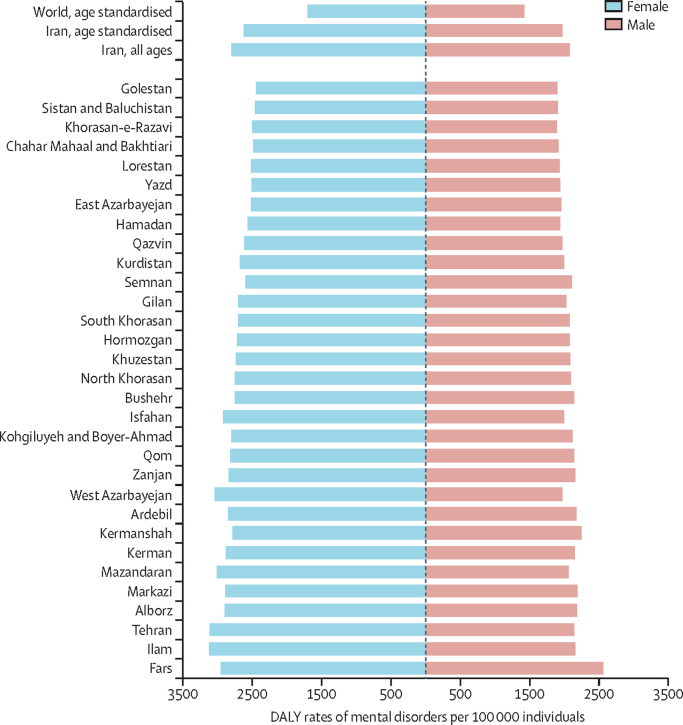
Age-standardised DALY rates of mental disorders in the provinces of Iran (2019) for males and females, compared with national and global levels DALYs=disability-adjusted life-years.

Figure 1 shows the DALY rates by sex in total and for each of the provinces. The female-to-male ratio of age-standardised burden ranged from 1·14 to 1·52 in different provinces, although only one of the provinces had a ratio lower than the global mean.

Considering the change in age composition of the population, age-standardised analysis would help in understanding the overall changes. The age-standardised DALY rate for mental disorders had a 1·8% increase between 1990 and 2019, although the UI includes zero (–4·1 to 7·7%; table 2). For most disorders, the change was between 0% and 5%, although eating disorders had a 25·9% increase and idiopathic developmental intellectual disability had a 41·3% decrease.

**Table 2:** Age-standardised DALY rates per 100 000 people

	**1990 DALY rate**	**2019 DALY rates**	**Percentage change, 1990–2019**
**Mental disorders**	**2255 (1670 to 2965)**	**2296 (1702 to 3034)**	**1·8% (−4·1 to 7·7%)**
Anxiety disorders	672 (470 to 922)	696 (483 to 955)	3·6% (−7·4 to 14·5%)
Attention-deficit hyperactivity disorder	32 (18 to 54)	32 (18 to 55)	2·9% (−47·6 to 53·3%)
Autism spectrum disorders	55 (36 to 81)	56 (37 to 82)	1·8% (−35·9 to 39·5%)
Bipolar disorder	174 (107 to 267)	176 (108 to 270)	1·3% (−20·0 to 22·5%)
Conduct disorder	75 (42 to 118)	73 (41 to 116)	−2·9% (−34·3 to 28·4%)
Depressive disorders	848 (576 to 1182)	890 (606 to 1248)	5·0% (−4·9 to 14·8%)
Eating disorders	43 (27 to 64)	54 (34 to 80)	25·9% (−24·6 to 76·3%)
Idiopathic developmental intellectual disability	95 (53 to 151)	56 (28 to 94)	−41·3% (−60·7 to −22·0%)
Other mental disorders	101 (65 to 153)	101 (64 to 153)	0·0% (−27·7 to 27·6%)
Schizophrenia	161 (118 to 203)	162 (118 to 206)	0·9% (−21·1 to 22·9%)

Data in parentheses are 95% uncertainty intervals. DALYs=disability-adjusted life-years.

**Figure 2:**
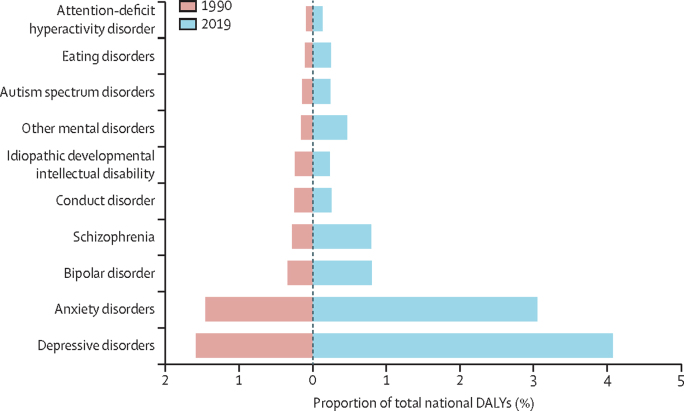
All-age DALYs for different mental disorders as a percentage of total national DALYs in 1990 and 2019 DALYs=disability-adjusted life-years.

The change in share of total national DALYs was not similar for different diseases between 1990 and 2019. Although no change in share was observed for conduct disorder and idiopathic developmental intellectual disability, noticeable increases were present in all-age DALY percentages of other disorders (figure 2).

Meanwhile, the overall prevalence of mental disorders has not changed much. The least prevalent disorder (ie, an eating disorder) had a noticeable increase, whereas the decrease in idiopathic developmental intellectual disability was also remarkable. The order of disorders based on prevalence has not changed between 1990 and 2019 (table 3).

**Table 3:** Prevalence of mental disorders per 100 000 people in 1990 and 2019 and the percentage change

	**1990 prevalence**	**2019 prevalence**	**Percentage change, 1990–2019**
**Mental disorders**	**18 251 (16 924 to 19 759)**	**18 016 (16 677 to 19 467)**	**–1·3% (−3·3 to 0·7%)**
Anxiety disorders	7036 (6019 to 8238)	7268 (6215 to 8502)	3·3% (0·1 to 6·7%)
Depressive disorders	4662 (4064 to 5345)	4861 (4240 to 5585)	4·3% (0·1 to 8·5%)
Attention-deficit hyperactivity disorder	2596 (1900 to 3514)	2665 (1948 to 3607)	2·7% (−2·9 to 8·2%)
Idiopathic developmental intellectual disability	2224 (1487 to 2983)	1394 (834 to 1981)	−37·3% (−41·5 to −33·1%)
Other mental disorders	1372 (1049 to 1738)	1369 (1047 to 1735)	−0·2% (−7·7 to 7·3%)
Bipolar disorder	808 (681 to 940)	817 (687 to 949)	1·1% (−8·7 to 10·9%)
Conduct disorder	615 (440 to 793)	596 (427 to 779)	−3·1% (−14·0 to 7·8%)
Autism spectrum disorders	364 (300 to 437)	370 (307 to 441)	1·6% (−13·1 to 16·4%)
Schizophrenia	252 (215 to 291)	254 (217 to 293)	0·8% (−16·8 to 18·4%)
Eating disorders	203 (149 to 263)	254 (190 to 328)	25·1% (2·0 to 48·2%)

Data in parentheses are 95% uncertainty intervals.

**Figure 3:**
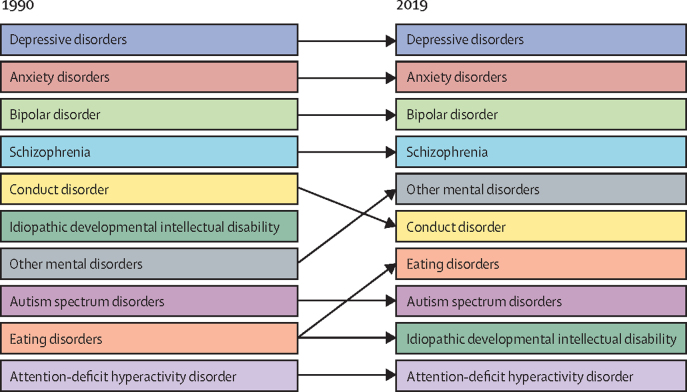
The change in the order of share of percentage for all-age DALYs for different mental disorders between 1990 and 2019 DALYs=disability-adjusted life-years.

Between 1990 and 2019, we observed a change in the share of DALYs for different mental disorders. The top four sources of DALYs (depressive disorders, anxiety disorders, bipolar disorders, and schizophrenia) caused 79% of DALYs in 1990 and 84% in 2019, with the same order of respective share. The incidence rate of mental disorders collectively showed a slight increase over the three decades, with a minor decrease in the final years of the period. This trend was primarily due to changes in the incidence of depressive disorder. Among other disorders, schizophrenia showed a relative increase, while the incidence of other disorders declined to varying degrees. (figure 3).

The subnational estimates of burden of disease are presented in the appendix (p 8). Although age-standardised DALYs at the national level showed a 1·8% increase, at the subnational level it ranged from –0·3% to 3·1% between 1990 and 2019, reflecting the diversity in population structure and changes in prevalence of mental health disorders within the country and at the provincial level. The general patterns of changes were similar at the subnational level, although the rates differed between provinces with a highest-to-lowest ratio of 1·22 for age-standardised DALY rates in 2019 (appendix p 8). The age-standardised DALY rates for different mental disorders in 1990 and 2019 are presented in the appendix (p 9).

## Discussion

The burden of mental disorders has increased in the world during the past three decades.^1^ Although the observed increase in the burden of mental disorders in Iran (77%) has been higher than the general increase in the world (55%),^1^ it has been lower than the WHO Eastern Mediterranean region countries (114%).^17^ The age-standardised DALYs have only slightly increased (1·8%), which means most of the increase is related to change in size and structure of the population. However, the share of mental disorders from total all-cause DALYs has substantially increased at both the national level (from 4·6% in 1990 to 10·3% in 2019) and across all provinces (appendix p 8). This rise has been faster in Iran (123%) than in the Eastern Mediterranean region (96%) and the world (58%). This increase has big implications from a public health perspective. There is an increasing need for mental health services across primary, secondary, and tertiary care levels. These mental health services across all care levels are particularly important because there is already a dearth of psychiatric and mental health services compared with the high level of actual needs for services.^18^

The female-to-male ratio of age-standardised DALY rates for mental disorders in Iran was higher than the global mean, at 1·34 compared with 1·19. The reasons for this finding need further research. However, the conflict between traditional gender roles and conservative societal expectations on the one side, and the needs and interests of the rapidly transforming population of Iranian women (especially from an educational perspective) on the other side, could have contributed to higher rates of anxiety and depressive disorders in women than men.

Iran has a national programme for integrating mental health services in primary health care that provides some basic mental health services throughout the country.^19^ Nevertheless, both case finding and service use are low in the system. Two-thirds of people with mental disorders do not receive any services for their mental health concerns. Barriers—such as a poorly understood need for care, little knowledge about mental disorders, discriminatory attitudes and stigmatisation of mental disorders, poor access to mental health services, and high costs—are challenges that need to be addressed.^20^, ^21^, ^22^, ^23^, ^24^

Considering the absolute and relative increase in the burden of mental disorders during the past 30 years, there is an urgent need to upgrade the related services both on a national level and regionally, by prioritising provinces that are in highest need. Both primary care and specialised services, as well as community-based and rehabilitative services, should be taken into consideration. We suggest strategies to promote not only preventive measures in society and primary care, but also case finding and specialised care—including inpatient services and, more importantly, aftercare and community-based services. Parts of the mental health services can be allocated to general practitioners and health-care workers who are not physicians. Health policy makers should invest much more in developing mental health services for the public, especially when there is local evidence of cost-effectiveness of such services.^25^, ^26^ It is also very important to address the social determinants of mental health to successfully reduce their burden.^27^ Policy makers should pay special attention to the determinants of mental health as a disease category that now constitutes more than 10% of the total burden of diseases in the country.

Possible reasons for this large increase in the share of mental disorders should be discussed. We suggest two possible reasons: first, because of the change in the population (appendix p 10), towards a population structure that is more susceptible to the most prevalent mental disorders (ie, depressive disorders and anxiety disorders); and second, the decrease or relatively slower increase in the burden of other diseases, particularly communicable diseases and injuries. The increase in the age-specific prevalence rates does not seem to be an important contributing factor during the studied period, because the age-standardised DALY rate has only slightly changed; however, there is evidence about the increasing rates of self-harm and suicidal ideations, especially in the highly educated population of Iran in the post-COVID-19 era, which is generally attributed to social and financial stressors.^28^, ^29^, ^30^

A similar pattern is seen for the burden of less prevalent but still important mental disorders, such as bipolar disorder, schizophrenia, and eating disorders. However, the burden of the disorders that are more prevalent during childhood and adolescence have not increased much or have even decreased to some extent, including attention-deficit hyperactivity disorder (ADHD), autism spectrum disorders, conduct disorder, and idiopathic developmental intellectual disability. A unique issue commonly observed in the southwestern Asian countries is the exceptionally high prevalence of consanguineous marriages, which has a great impact on composition and prevalence of mental disorders. During the past few decades, with the revolutionary developments in clinical genetics (including next-generation sequencing and prenatal diagnostics), the diagnosis of congenital genetic disorders was enormously enhanced. These developments can potentially account for at least part of the decrease in the prevalence and age-standardised DALYs of idiopathic developmental intellectual disability during the 1990–2019 period. However, the effects of the efforts by the Iranian political establishment to increase the population through restricting the access to contraceptives, prenatal screening, and subsequent abortion are elusive. The burdens of autism spectrum disorders and ADHD have a small to moderate increase that could be partly due to the changes in diagnostic criteria made in the Diagnostic and Statistical Manual of Mental Disorders (DSM) volume 5. Although all the definitions have been standardised during the GBD analysis process, and data have been crosswalked and adjusted to the standard definition even for the previous years, this might not completely preclude the influence of updated criteria. Autism spectrum disorder is a new diagnosis in DSM-5, which has been formed by integrating previously distinct diagnoses. Furthermore, the DSM-5 permits a simultaneous diagnosis of autism spectrum disorders and ADHD that might affect the prevalence of the disorders.

At the provincial level, the change in age-standardised DALYs varied from –0·3% to 3·1% (appendix p 8), which shows a small but increasing trend in most of the provinces. This trend is in line with the previous finding that the overall age-standardised DALYs of mental disorders has not changed much in the country. However, the total amount of mental health DALYs has notably increased, reaching as high as 569% in the Zanjan province and 393% in Gilan province. This variation might have mainly resulted from a non-uniform change in population composition in different provinces.

The Iranian population has undergone different social and economic stressors during the past few decades. A combination of non-effective governmental economic strategies and multiple international sanctions have negatively affected purchasing power of the people and exposed many individuals to long-term financial stressors. However, the relationship between mental disorders and economic problems is not linear, and there is a long list of variables at the individual level or community level that might influence this association.^31^ Despite some evidence of deteriorated mental health status during sanctions in Iran^32^ and a negative effect of sanctions on the access to medical services and medications,^33^ we did not observe a significant increase in the age-standardised burden of mental disorders. The reason for this absence of association should be further assessed in other studies. It is possible that living in unstable and stressful situations for several decades might have produced some kind of resilience to buffer the effect of new stressors, such as sanctions. Additionally, it might be due to the modelling approach that cannot catch all the epidemiological changes when relevant external factors (eg, sanctions) are not used as input data. Other possible reasons need to be addressed in future studies.

Eating disorders had a large increase (26%) in the age-standardised DALY rate from 1990 to 2019. This increase could be understood in the light of the explosive increase in the access to internet, satellite channels, and social media and adopting the globalised beauty standards of leanness, which are highly publicised in the media. This increase is especially important because the clinicians and health-care facilities in Iran are not generally familiar with eating disorders and therefore are not well prepared to deal with it. This increased prevalence heralds a need for the development of related services in the health-care delivery system, as well as development and implementation of primary and secondary preventive programmes for eating disorders.

There were a few limitations to this subanalysis. Access to high quality data on epidemiological measures of mental disorders is a major issue in Iran, as with many other countries in the region. Although there were several mental health surveys in Iran during the past decades, some of them used non-standard methods or non-representative samples that made them unsuitable for GBD models. However, GBD methodology of Bayesian meta-regression modelling reduces the issue, with a transparent approach to generate a set of internally consistent data. The findings might also have been affected by the underlying biases despite the best efforts to adjust for them.

Although there is a comprehensive understanding about the dependent comorbidities between mental disorders, we did not include them in the analysis. Addressing all possible combinations of dependent comorbidities in the GBD poses a major challenge, especially because of the computational complexities involved. There is a plan to take a new approach by concentrating on quantifying prevalent comorbidities and targeting a subset of comorbid pairs for correction in the future rounds of GBD.

In conclusion, our findings highlight the increase in crude DALYs and their share of the disease burden for mental disorders both at the national and provincial levels and this requires attention from different groups of policy makers. The findings call for revisiting supportive social and economic policies to improve welfare and equity that might affect the mental health of Iranian people directly or indirectly, as well as health sector policies targeting access to high-quality and efficient mental health services. The results of the study shed light on the strategy and priorities in the field of mental health at national and provincial levels. Such evidence is especially important for a country with a large young population group and severe economic challenges, as priority setting is a crucial step considering the scarce resources.

### Iran Subnational Mental Health GBD Collaborators

### Affiliations

## Contributors

## Data sharing

To download the data used in these analyses, please visit the Global Health Data Exchange at https://ghdx.healthdata.org/gbd-2019.

## Declaration of interests

We declare no competing interests.
