# Supplementary material for: National and subnational burden of mental disorders in Iran (1990–2019): findings of the Global Burden of Disease 2019 study
Source: Lancet Glob Health. 2024 Nov 20;12(12):e1984–92. doi: 10.1016/S2214-109X(24)00342-5 (PMC11584315; doi:10.1016/S2214-109X(24)00342-5)
Supplement: Supplementary appendix [file mmc1.pdf]

THE LANCET
Global Health

Supplementary appendix

This appendix formed part of the original submission and has been peer reviewed. We post it as supplied by the authors.

Supplement to: Iran Subnational Mental Health GBD Collaborators. National and subnational burden of mental disorders in Iran (1990–2019): findings of the Global Burden of Disease 2019 study. *Lancet Glob Health* 2024; **12:** e1984–92.

Supplement Material.

Contents

Table S1. Cause list of mental disorders and their ICD-10 codes in the Global Burden of Disease 2019 2

Table S2: Classification codes, disability weights, prior assumptions and male-to-female ratio for sex-splitting of input data for mental disorders (Global Burden of Diseases 2019)........ 3

Table S3: Location-level and study-level covariates used for modeling of mental disorders (Global Burden of Disease 2019)........ 5

Table S4. Age standardized DALY (per 100,000 population) and All age DALY share of Mental Disorders for provinces of Iran 8

Table S1. Cause list of mental disorders and their ICD-10 codes in the Global Burden of Disease 2019

Cause name (Cause level)	DSM-IV-TR	ICD-10 codes
Mental disorders (2)		
Schizophrenia (3, 4)	295.10-295.30, 295.60, 295.90	F20-F20.9, F25-F25.9
Depressive disorders (3)		F32-F33.9, F34.1
- Major depressive disorder (4)	296.21–24, 296.31–34	F32-F33.9
- Dysthymia (4)	300.4	F34.1
Bipolar disorder (3, 4)	296.0–296.7, 296.89, 301.13	F30-F31.9, F34.0
Anxiety disorders (3, 4)	300.0-300.3, 208.3, 309.21, 309.81	F40-F44.9, F93-F93.2
Eating disorders (3)		F50-F50.9
- Anorexia nervosa (4)	307.1	F50.0-F50.1
- Bulimia nervosa (4)	307.51	F50.2-F50.5
Autism spectrum disorders (3, 4)	299.00, 299.80, 299.8, 299.8, 299.10	F84.0, F84.1, F84.2, F84.3, F84.4, F84.5, F84.8, F84.9
Attention-deficit/hyperactivity disorder (3, 4)	314.0, 314.01	F90-F90.9
Conduct disorder (3, 4)	312.81-312.89	F91-F92.9
Idiopathic developmental intellectual disability (3, 4)	317, 318.0, 318.1, 318.2, 319<br>[from any unknown source after the prevalence of all other sources of Intellectual Disability is accounted for]	F70-F79.9, Z81.0
Other mental disorders (3, 4)	300.3, 301.0; 301.2, 301.22, 301.5–301.9	F04-F06.1, F06.3-F07.0, F08-F09.9, F21-F24, F26-F29.9, F34, F34.8-F34.9, F38-F39, F45-F49, F51-F52.9, F55-F55.8, F56-F69.0, F80-F89.0, F93.3-F99.0, G47-G47.29, G47.4-G47.9, R40-R40.4, R45-R46.89, R55-R55.0, Z03.2, Z04.6-Z04.72, Z13.4, Z64, Z81, Z81.8, Z86.5-Z86.59
DSM: Diagnostic and Statistical Manual of Mental Disorders; ICD: International Classification of Diseases		

Table S2: Classification codes, disability weights, prior assumptions and male-to-female ratio for sex-splitting of input data for mental disorders (Global Burden of Diseases 2019)

Disorder	Classification	Estimated M:F ratio (%95 UI) for Prevalence sex-split	Disability Weight	Prior(s) / Assumptions
**Schizophrenia**	DSM-IV-TR: 295.10-295.30, 295.60, 295.90; ICD 10: F20	1.17 (0.60 – 1.75)	Acute state: 0.778(0.606 – 0.9), Residual state: 0.588(0.411 – 0.754)	No incidence before age 10 and after age 80; Remission was set to a maximum of 0.04
**Major depressive Disorder**	DSM-IV-TR: 296.21–24, 296.31–34; ICD-10: F32.0–9, F33.0–9; excluding those cases due to a general medical condition or substance induced cases	0.52 (0.26 – 0.77)	Mild: 0.145 (0.099 – 0.209), Moderate: 0.396 (0.267 – 0.531), Severe: 0.658 (0.477 – 0.807)	No incidence and prevalence before age 3; an average remission rate: 1.45 (1.3–1.6); average duration: 0.65 (0.59–0.70) of a year
**Dysthymia**	DSM-IV-TR: 300.4, ICD-10: F34.1; excluding those cases due to a general medical condition or substance-induced cases	0.66 (0.50 – 0.83)	Symptomatic: 0.145 (0.099 – 0.209)	No incidence and prevalence before age 3; Excess-mortality was set to 0
**Bipolar Disorder**	DSM-IV-TR: 296.0–296.7, 296.89, 301.13; ICD-10: F30.0-F30.9, F31.0–F31.6, F31.8–F31.9, F34.0. Excluded were bipolar disorder due to a general medical condition or substance-induced cases.	0.82 (0.42 – 1.22)	Manic: 0.492 (0.341 – 0.646), Depressive: 0.396 (0.267 – 0.531), Residual: 0.032 (0.018 – 0.051)	No incidence and prevalence before age 10; Remission was set to a maximum of 0.05
**Anorexia nervosa**	DSM-IV-TR: 307.1; ICD-10: F50.0-50.1	0.24 (0.05 – 0.43)	0.224 (0.150–0.312)	No incidence before age 5 or 50 years onward; A cap of 0.6 was placed on remission
**Anxiety Disorders**	DSMIV-TR: 300.0-300.3, 208.3, 309.21, 309.81; ICD-10: F40-42, F43.0, F43.1, F93.0-93.2, F93.8. Excluded were anxiety disorders due to a general medical condition and substance-induced anxiety disorder.	0.55 (0.38 – 0.72)	Mild: 0.03 (0.018 – 0.046), Moderate: 0.133 (0.091 – 0.186), Severe: 0.523 (0.362 – 0.677)	No incidence and prevalence before age 2 and after age 95; Remission was set to a maximum of 0.2
**Bulimia nervosa**	DSM-IV-TR: 307.51; ICD-10: F50.2	0.37 (0.26-0.47)	0.223 (0.149 – 0.311)	No incidence prior to 10 years of age or onward from 40 years of age

**Autism spectrum disorders**	DSM-IV-TR: Autistic disorder (299.00), Pervasive Developmental Disorder, Pervasive Developmental Disorder Not Otherwise Specified (299.80), Rett's disorder (299.8), Asperger's Disorder (299.8) and Childhood Disintegrative Disorder (299.10); ICD-10: Childhood autism (F84.0), Atypical autism (F84.1), Rett syndrome (F84.2), Other childhood disintegrative disorder (F84.3), Overactive disorder associated with mental retardation and stereotyped movements (F84.4), Asperger syndrome (F84.5), Other pervasive developmental disorders (F84.8), and Pervasive disorder unspecified (F84.9)	4.39 (3.36 – 5.41)	ASD without ID: 0.143 (0.094 – 0.202),<br>ASD with borderline ID: 0.152 (0.103 – 0.212),<br>ASD with mild ID 0.179 (0.125 – 0.245),<br>ASD with moderate ID: 0.228 (0.160 – 0.310),<br>ASD with severe ID: 0.279 (0.195 – 0.378),<br>ASD with profound ID: 0.313 (0.215 – 0.422),	All incidence of ASD occurs at birth; Remission was set to 0
**Attention-deficit/hyperactivity disorder**	DSM-IV-TR: 314.0, 314.01; ICD-10: F90	2.52 (0.57 – 4.46)	0.045 (0.028–0.066)	No incidence prior to 3 years of age or onward from 12 years of age; Remission was set to zero prior to 12 years; Excess mortality was set to zero
**Conduct disorder**	DSM-IV-TR: 312.81-312.89; ICD-10: F91	2.31 (0.73 – 3.88)	0.241 (0.159–0.341)	No incidence or prevalence prior to 5 years of age or after 18 years of age; Excess mortality was set to zero; Remission and incidence were capped between ages 4 and 17 years
**Other mental disorders**	DSM-IV-TR: 300.3, 301.0; 301.2, 301.22, 301.5–301.9; ICD-10: F60		Mild: 0.03 (0.018 – 0.046),<br>Moderate: 0.133 (0.091 – 0.186),<br>Severe: 0.523 (0.362 – 0.677)	No incidence and prevalence before age 14; Remission was set to a maximum of 0.01; Excess mortality was set to 0; A restriction on location random effects of -0.1 to 0.1 for prevalence
**Developmental intellectual disability**	DSM-IV-TR: 317, 318.0, 318.1, 318.2, 319; ICD-10: F70-F79.9, Z81.0	0.90 (0.87 to 0.93)	Borderline: 0.011 (0.005–0.02),<br>Mild: 0.043 (0.026–0.064),<br>Moderate: 0.10 (0.066–0.142,<br>Severe: 0.16 (0.107–0.226),<br>Profound: 0.20 (0.133–0.283)	N/A

Table S3: Location-level and study-level covariates used for modeling of mental disorders (Global Burden of Disease 2019)

Disorder	Covariate	Type	Parameter	Reference	Exponentiated Beta (%95 UI)	Beta coefficient, Log (%95UI)	Adjustment factor	Gamma
**Schizophrenia**	LDI (Per capita $US)	Location-level	Excess Mortality	N/A	0.58 (0.37 – 0.90)	N/A	N/A	N/A
**Major Depressive Disorder**	Alternative: Past year prevalence	Study-level	Prevalence	Reference: Past month/point prevalence, from a diagnostic tool, administered by a clinician	N/A	0.69 (-0.20 – 1.57)	1.99 (0.82 – 4.79)	0.43
**Major Depressive Disorder**	Alternative: Symptom scale	Study-level	Prevalence	Reference: Past month/point prevalence, from a diagnostic tool, administered by a clinician	N/A	1.00 (0.10 – 1.88)	2.71 (1.11 – 6.56)	0.43
**Major Depressive Disorder**	Alternative: World Health Survey	Study-level	Prevalence	Reference: Past month/point prevalence, from a diagnostic tool, administered by a clinician	N/A	0.68 (-0.22 – 1.57)	1.98 (0.80 – 4.83)	0.43
**Major Depressive Disorder**	Alternative: Lay-interviewer	Study-level	Prevalence	Reference: Past month/point prevalence, from a diagnostic tool, administered by a clinician	N/A	-0.22 (-1.08 – 0.65)	0.79 (0.34 – 1.91)	0.43
**Major Depressive Disorder**	Mean war mortality rate in the previous 10 years	Location-level	Prevalence	N/A	1.63 (1.07 – 2.53)	N/A	N/A	N/A
**Major Depressive Disorder**	Log-transformed age-standardized SEV scalar: Depression	Location-level	Prevalence	N/A	3.27 (2.97 – 3.48)	N/A	N/A	N/A
**Major Depressive Disorder**	Gallup: Negative experience index Location-level Prevalence	Location-level	Prevalence	N/A	1.01 (1.00 — 1.04)	N/A	N/A	N/A
**Dysthymia**	Alternative: lay interviewer	Study-level	Prevalence	Reference: clinical diagnosis	N/A	0.22 (-1.08 – 0.68)	0.80 (0.34 – 1.97)	0.43

**Bipolar Disorder**	Alternative: point or past month prevalence	Study-level	Prevalence	Reference: past year or 12-month prevalence of bipolar disorder	N/A	0.45 (-0.02 – 0.92)	1.57 (0.98 – 2.50)	0.23
**Bipolar Disorder**	Alternative: lifetime prevalence	Study-level	Prevalence	Reference: past year or 12-month prevalence of bipolar disorder	N/A	-0.37 (-0.85 – 0.10)	0.69 (0.43 – 1.11)	0.23
**Anorexia nervosa**	LDI (Per capita $US)	Location-level	Prevalence	N/A	1.48 (1.26 — 1.64)	N/A	N/A	N/A
**Anorexia nervosa**	LDI (Per capita $US)	Location-level	Excess Mortality	N/A	0.79 (0.66 — 0.90)	N/A	N/A	N/A
**Anxiety Disorders**	Alternative: past year prevalence	Study-level	Prevalence	Reference: past month or point prevalence	N/A	0.46 (0.01 – 0.91)	1.58 (0.99 – 2.41)	0.23
**Anxiety Disorders**	Mean war mortality rate in the previous 10 years	Location-level	Prevalence	N/A	1.65 (1.07 — 2.54)	N/A	N/A	N/A
**Anxiety Disorders**	Gallup: Negative experience index	Location-level	Prevalence	N/A	2.48 (1.80 — 3.61)	N/A	N/A	N/A
**Bulimia Nervosa**	No cross-walk							
**Bulimia Nervosa**	LDI ($ per capita)	Location-level	Prevalence	N/A	1.54 (1.38 — 1.64)	N/A	N/A	N/A
**Bulimia Nervosa**	LDI ($ per capita)	Location-level	Excess Mortality	N/A	0.75 (0.63 — 0.88)	N/A	N/A	N/A
**Autism spectrum disorders**	Alternative: Estimate represents autism (rather than ASD)	Study-level	Prevalence	Reference: Estimate represents ASD from general population surveys, with additional case finding or total population screening	N/A	-0.93 (-1.49 – -0.36)	0.40 (0.23 – 0.70)	0.29
**Autism spectrum disorders**	Alternative: General population survey without additional case finding	Study-level	Prevalence	Reference: Estimate represents ASD from general population surveys, with additional case finding or total population screening	N/A	-0.29 (-0.91 – 0.33)	0.75 (0.40 – 1.39)	0.29
**Autism spectrum disorders**	Alternative: Record report	Study-level	Prevalence	Reference: Estimate represents ASD from general population surveys, with additional case finding or total population screening	N/A	-0.17 (-0.74 – 0.41)	0.85 (0.48 – 1.50)	0.29

**Autism spectrum disorders**	Alternative: Review of record notes	Study-level	Prevalence	Reference: Estimate represents ASD from general population surveys, with additional case finding or total population screening	N/A	0.22 (-0.40 – 0.83)	1.24 (0.67 – 2.30)	0.29
**Attention-deficit/hyperactivity disorder**	No cross-walk							
**Conduct disorder**	No cross-walk							
**Other Mental Disorders**	US National Epidemiological Survey on Alcohol and Related Conditions (NESARC)	Study-level	Prevalence	Australian National Survey of Mental Health and Wellbeing ofAdults (NSMHWB)	N/A	N/A	2.04 (1.82 – 2.34)	N/A
**Developmental Intellectual Disability**	LDI ($ per capita)	Location-level	Prevalence	N/A	0.69 (0.63 to 0.76)	N/A	N/A	N/A
**Developmental Intellectual Disability**	Age- and sex-specific SEV for child underweight	Location-level	Prevalence	N/A	4.42 (1.20 to 15.99)	N/A	N/A	N/A

Table S4. Age standardized DALY (per 100,000 population) and All age DALY share of Mental Disorders for provinces of Iran

	Age standardized DALY of			All age DALY share of Mental		
	1990	2019	%	1990	2019	% Change
**Iran (Islamic Republic of)**	2255	2296	**1.8%**	0.046	0.103	**123%**
Alborz	2277	2347	**3.1%**	0.061	0.114	**86%**
Ardebil	2297	2339	**1.8%**	0.041	0.100	**142%**
Bushehr	2297	2331	**1.5%**	0.052	0.114	**121%**
Chahar Mahaal and Bakhtiari	2085	2108	**1.1%**	0.049	0.107	**118%**
East Azarbayejan	2053	2095	**2.1%**	0.043	0.088	**103%**
Fars	2527	2547	**0.8%**	0.061	0.106	**75%**
Gilan	2116	2149	**1.6%**	0.018	0.087	**393%**
Golestan	2055	2094	**1.9%**	0.041	0.085	**108%**
Hamadan	2088	2104	**0.8%**	0.041	0.082	**101%**
Hormozgan	2288	2340	**2.3%**	0.041	0.110	**169%**
Ilam	2370	2417	**2.0%**	0.049	0.109	**122%**
Isfahan	2210	2245	**1.6%**	0.066	0.104	**57%**
Kerman	2420	2427	**0.3%**	0.050	0.104	**107%**
Kermanshah	2308	2330	**0.9%**	0.044	0.092	**111%**
Khorasan-e-Razavi	2119	2119	**0.0%**	0.036	0.090	**151%**
Khuzestan	2278	2338	**2.6%**	0.052	0.101	**95%**
Kohgiluyeh and Boyer-Ahmad	2294	2344	**2.2%**	0.045	0.112	**149%**
Kurdistan	2187	2180	**-0.3%**	0.035	0.098	**178%**
Lorestan	2066	2097	**1.5%**	0.045	0.095	**112%**
Markazi	2304	2345	**1.8%**	0.052	0.102	**94%**
Mazandaran	2280	2305	**1.1%**	0.068	0.099	**45%**
North Khorasan	2323	2344	**0.9%**	0.036	0.096	**169%**
Qazvin	2116	2124	**0.4%**	0.050	0.100	**101%**
Qom	2274	2343	**3.0%**	0.059	0.120	**105%**
Semnan	2150	2164	**0.6%**	0.054	0.101	**86%**
Sistan and Baluchistan	2300	2350	**2.2%**	0.033	0.090	**175%**
South Khorasan	2337	2347	**0.4%**	0.037	0.102	**173%**
Tehran	2350	2391	**1.7%**	0.089	0.129	**44%**
West Azarbayejan	2385	2409	**1.0%**	0.046	0.104	**127%**
Yazd	2091	2130	**1.8%**	0.055	0.100	**82%**
Zanjan	2286	2342	**2.5%**	0.016	0.108	**569%**

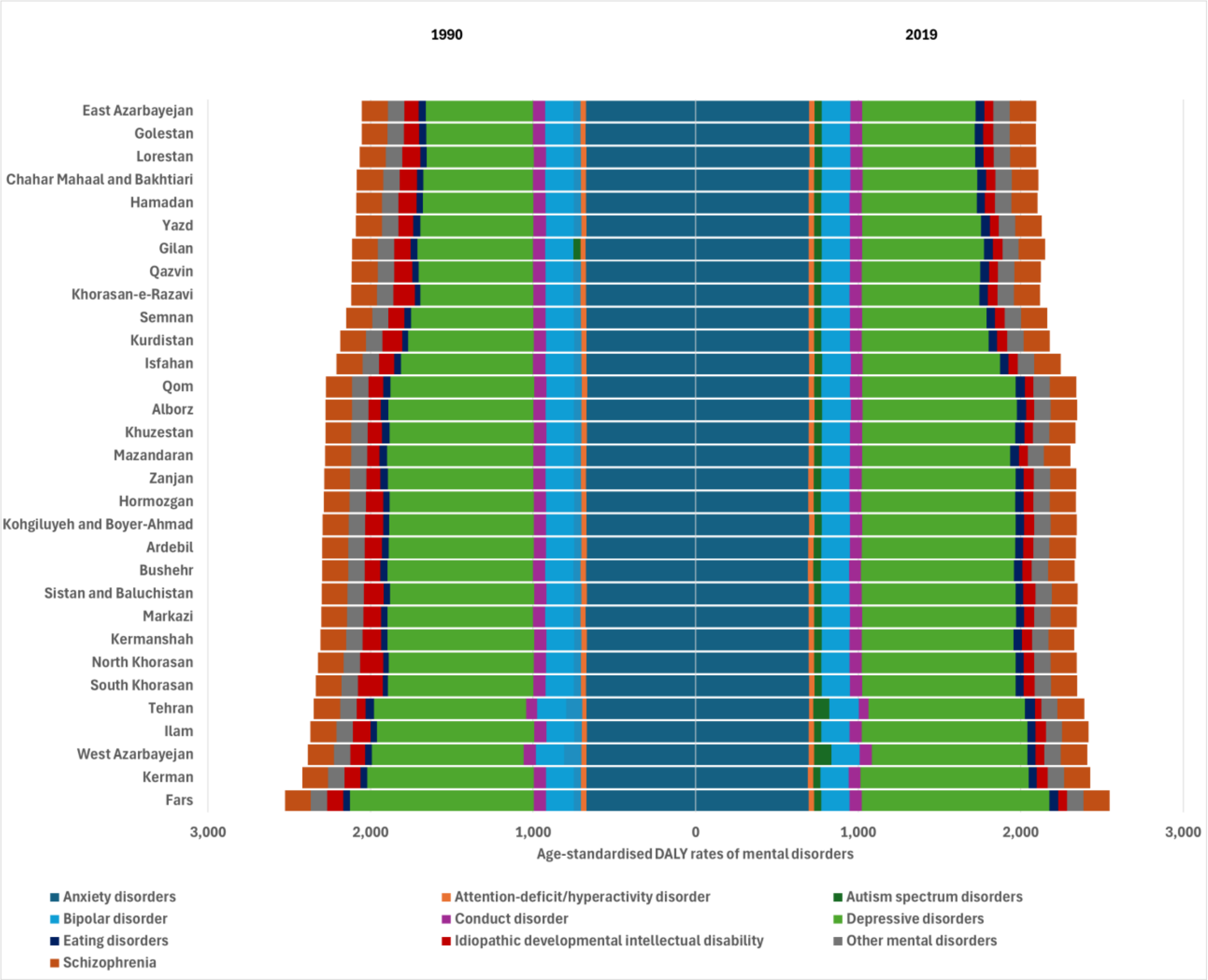

Figure S1. Changes in age-standardised DALY rates of each mental disorder in provinces of Iran between 1990 and 2019

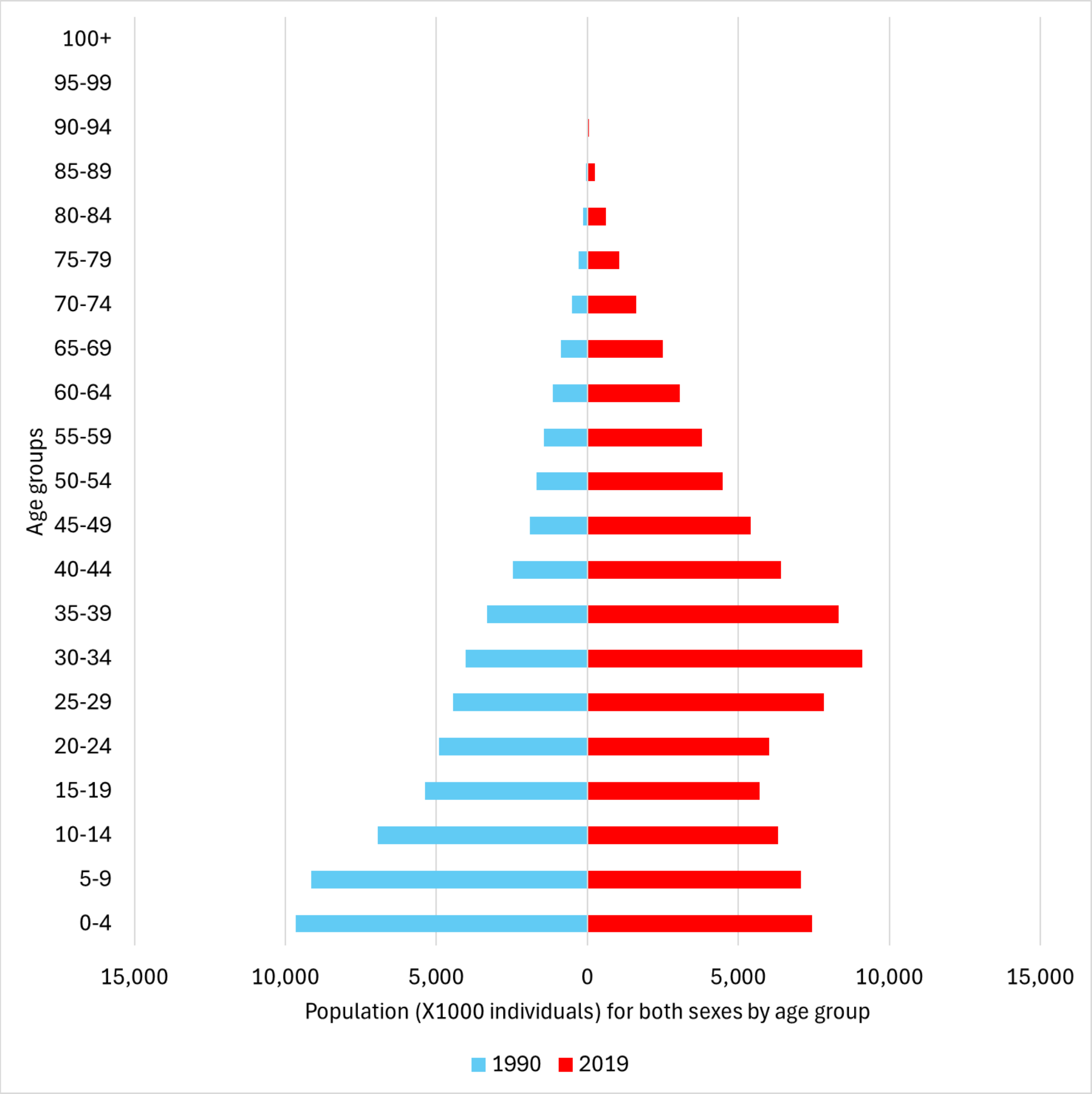

Figure S2. Age pattern of the population in Iran in 1990 compared to 2019
